# The effectiveness of inspections on reported mosquito larval habitats in households: A case-control study

**DOI:** 10.1371/journal.pntd.0007492

**Published:** 2019-06-26

**Authors:** Joel Aik, Zhi Wei Neo, Jayanthi Rajarethinam, Kaiyun Chio, Wing Mun Lam, Lee-Ching Ng

**Affiliations:** 1 Environmental Health Institute, National Environment Agency, Singapore; 2 School of Public Health and Community Medicine, Faculty of Medicine, University of New South Wales, New South Wales, Australia; 3 Environmental Public Health Operations Department, National Environment Agency, Singapore; Centers for Disease Control and Prevention, Puerto Rico, UNITED STATES

## Abstract

**Background:**

Dengue is an arboviral disease that imposes substantial health and economic burdens across the globe. Vector control remains a key strategy in settings where Dengvaxia (a dengue vaccine) has not been licenced due to safety concerns and where mass immunization programmes are not cost-effective. Though inspections are used as part of arboviral disease control programmes, evidence of their impact on the entomological activity in households is sparse.

**Methodology/Principal findings:**

We analysed nationally representative household inspection data collected from Singapore over a 3-year period, to determine the effect of inspections on reported mosquito larval habitats in households. A case was a household with a positive report of a mosquito larval habitat in its most recent inspection in 2017. A control was a household that was reported free of mosquito larvae in its most recent inspection in 2017. Using multivariable logistic regression, we analysed 3,205 cases and 557,044 controls. Households averaging three inspections per annum were associated with reduced odds of mosquito larval habitat reports [Adjusted Odds Ratio (AOR): 0.49, 95% Confidence Interval (95% CI): 0.38 to 0.63]. The effect of inspections declined with decreasing inspection frequencies but remained protective at lower levels. Longer intervals (30 to 36 months) between the most recent two successive inspections were associated with increased odds of mosquito larval habitat reports (AOR: 1.28, 95% CI: 1.06 to 1.56) compared to those carried out less than 6 months apart. Mosquito larval habitat reports exhibited a dependence on spatial and household-level characteristics such as the location of the community district, housing type and housing floor level. We observed a four-fold increase in the odds of mosquito larval habitat reports in households with an immediate previous report of larval activity compared to those that did not have one (AOR: 4.52, 95% CI: 3.67 to 5.56).

**Conclusions/Significance:**

Our study confirms the protective effect of inspections on reported mosquito larval habitat reporting in households. Spatial, temporal and household-level characteristics should be accounted for in prioritizing vector control resources. Alternative strategies may help address recurrent entomological activity in households.

## Author summary

Dengue is a disease that is transmitted by *Aedes* mosquitoes. In areas where the use of a dengue vaccine has not been authorized due to safety concerns and is not cost-effective, reducing the population of *Aedes* mosquitoes remains the key method in controlling the disease. One approach to mosquito population control is the use of inspections to identify and eliminate mosquito larval habitats in homes. However, evidence supporting the use of this approach is limited. In this study, which used national data from Singapore, we investigated the relationship between the frequency of inspections and the propensity for mosquito larval habitat reports in homes. We found that homes that had been inspected more frequently had fewer reports of mosquito larval habitats. We also found that the characteristics and locations of homes, and their history of previously reported mosquito larval habitats influenced present findings for mosquito larval habitats. We conclude that homes that are more frequently inspected have a lower number of reported mosquito larval habitats. Knowledge of the characteristics and locations of homes, and their history of previous reports may improve resource allocation for the control of mosquitoes in homes.

## Introduction

Dengue is an arboviral disease that imposes a substantial burden on economic development and human health across the globe. The annual global economic cost of this vector-borne disease has been estimated at USD$8.9 billion [1]. Global estimates place the annual number of dengue infections at 390 million [2] and deaths at nearly 10,000 [3]. The health impact of dengue is enormous yet disproportionate, with the highest incidence occurring in Asia [2]. In 2017, the World Health Organization (WHO) set ambitious goals for the global reduction of at least 75% and 60% in vector-borne disease mortality and morbidity respectively by 2030 [4]. To reach these goals, public health services will need to accelerate the application of effective interventions.

A vaccine would represent an important advancement in controlling dengue, especially in the tropics and sub-tropics where the disease is common. Ongoing clinical trials evaluating the tetravalent dengue vaccine candidates include those developed by Takeda (TAK-003) and the National Institute of Allergy and Infectious Diseases (TV-003/TV-005). Although the Dengvaxia vaccine (CYD-TDV) which was developed by Sanofi-Pasteur and first registered in 2015 demonstrated intermediate efficacy [5], the dengue seronegative recipients of this vaccine experienced a higher risk of severe dengue symptoms [6]. To ensure safer health outcomes for Dengvaxia vaccine recipients, pre-immunisation screening is highly recommended [7, 8] but this may add to the burden of resources required for mass immunization programmes.

Dengue vaccines may hold the promise of disease relief for dengue endemic countries that have the necessary resources to implement and sustain immunization programmes. However, in settings where Dengvaxia has not been licenced due to safety concerns and mass immunization programmes are not cost-effective, vector control remains a key strategy in mitigating the impact of dengue transmission. The use of both immunization and vector control strategies may be required to control the disease more effectively [9].

Dengue infections are driven primarily by the *Aedes aegypti* mosquito [10], though the *Aedes albopictus* mosquito also plays an important role [11]. Besides dengue, *Aedes* mosquitoes are also vectors for Chikungunya, Zika and Yellow Fever [12]; therefore interventions aimed at their reduction are protective for multiple diseases. Integrated Vector Management (IVM) is a rational decision-making process to optimize the use of resources and is the WHO’s preferred approach to improving vector control [13]. An important part of IVM is strengthening the evidence for setting-specific public health interventions in order to inform decisions on vector control and resource allocation [14]. One systematic review of cluster-randomized controlled trials aimed at controlling the *Aedes aegypti* mosquito reported that community mobilization and participation interventions were effective in reducing entomological indices but not those that relied on chemical control [15]. The meta-analysis from another systematic review reported that (i) window and door screens and (ii) community-based environmental management and water container covers, were both effective in reducing the risk of dengue transmission [16]. These studies estimated the effects of interventions over a baseline of ongoing government vector control programmes but none estimated the independent effect of household inspections on dengue transmission or entomological activity. Systematic reviews have highlighted the paucity of evidence for the effectiveness of *Aedes* vector control interventions [16–18]. Strengthening the evidence for vector control interventions is thus necessary to improve arboviral control policy and practice.

Inspections devoted to the elimination of mosquito larval habitats are also part of the arboviral control programmes in places such as Queensland (Australia), Florida (United States of America), Taiwan and Singapore [19], as well as in most other dengue endemic countries. However, little is known about the impact of such inspections on entomological outcomes in households. In this study, we examined the effectiveness of inspections on the number of reported mosquito larval habitats in homes in Singapore, over a 3-year period.

## Methods

### Ethics statement

This study was granted approval by the Environmental Health Institute of the National Environment Agency, Singapore (TS231). The study did not involve human participants.

### Study setting

Located within Southeast-Asia, Singapore is a city-state with a land area of 719 km^2^ and an estimated population of 5.6 million [20]. Singapore experiences a tropical climate all year round, with ambient air temperature usually reaching a peak in the middle of the calendar year and rainfall occurring on almost half of all calendar days [21]. Dengue is endemic in this country and exhibits a cyclical epidemic trend characterized by the switching of dengue virus serotypes 1 and 2 [22].

In Singapore, apartment blocks are the most common housing type, accounting for 95% of homes occupied by residents [23]. These blocks may have as few as 3-storeys or as many as 50-storeys, with the majority between the 10- and 30-storey range. Apartment blocks built by the government (known as “public apartments”) are the majority of all residential housing while those built by the private sector (known as “private apartments”) are the minority. In general, public apartments are more affordable compared to private apartments. A small proportion of residents live in landed houses built by the private sector and these are generally the least affordable among the three types of housing. It is common to observe plants and artificial containers in the external paved and turf areas of landed houses. All homes in Singapore are linked to a national piped drinking water network and have access to daily garbage removal services coordinated either by the state or municipal authorities.

**Fig 1:**
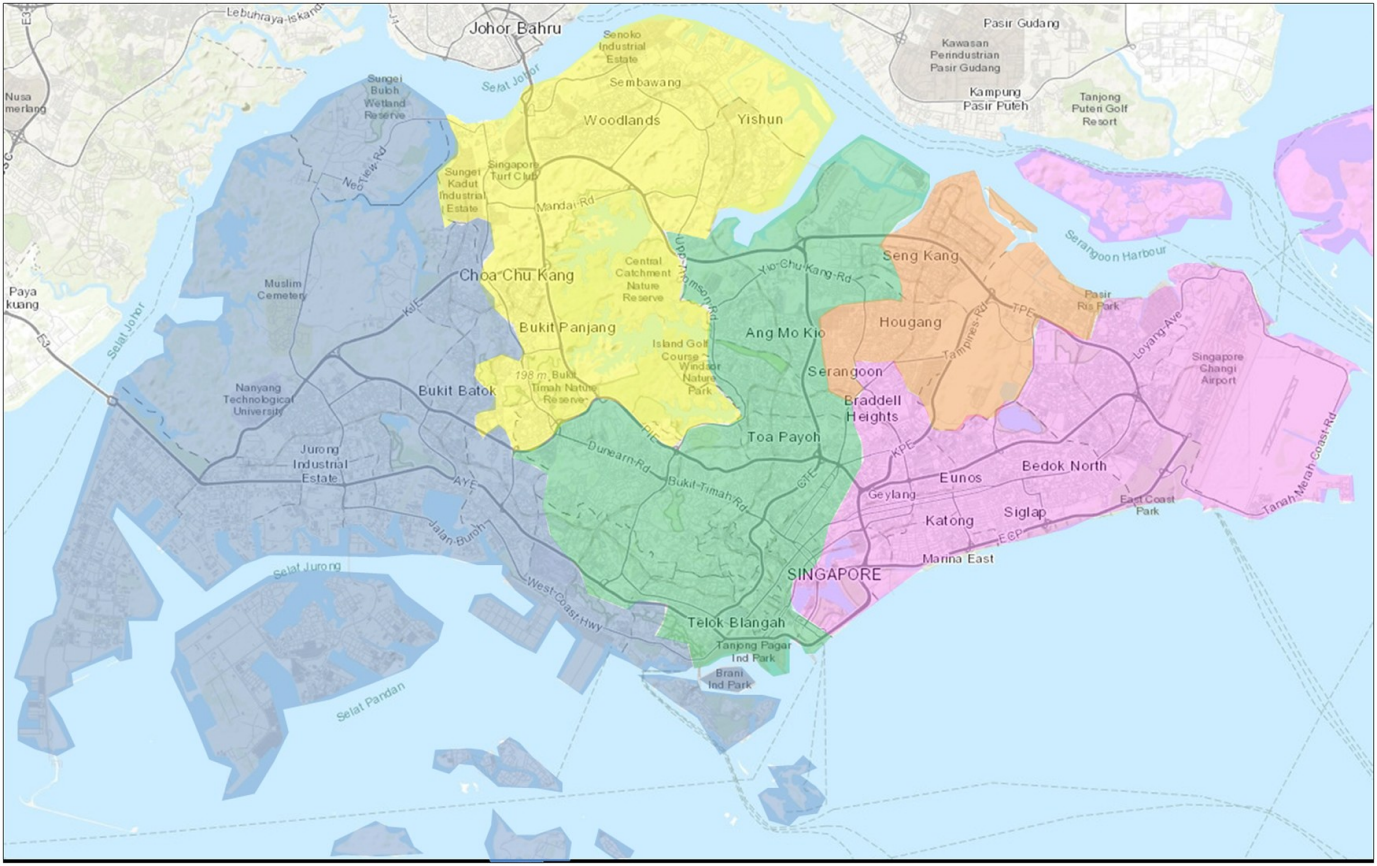
Public health inspectorate operating boundaries in Singapore, 2018. The coloured spaces depict the operating boundaries of the National Environment Agency’s five inspectorates in 2018.

The National Environment Agency (NEA) is responsible for implementing the national arboviral control strategy in Singapore. In each of the five community districts (see Fig 1), inspectors from the NEA’s five corresponding public health inspectorates conduct entomological surveillance and disease control activities in residential and non-residential premises [24]. The inspectors carry out household inspections in the presence of the occupant(s) and communicate their findings and recommendations for mosquito and disease prevention. More than 1 million inspections for mosquito breeding are performed each year. Pupae and larvae collected from natural and artificial containers during inspections are sent for independent entomological identification to the Environmental Health Institute. Common mosquito habitats found in homes include pails, flower pot plates and trays, vases, hardened soil and plant axils [25]. Standing water from containers positive for mosquito larvae are treated by inspectors with Temephos to ensure that none of the mosquito immatures emerge as adults. Punitive fines for the detection of entomologically identified mosquito larval habitats in households are set at S$200 [26], though the law allows for a higher quantum at the Director-General for Public Health’s discretion [27]. The law also allows for entry into inaccessible premises without the consent of the owner if the risk for mosquito breeding is high [27], and such inspections may be carried out in the absence of the occupants, though these are infrequent.

### Study participants and design

Our main study question was “Are the reported mosquito larval habitats in households associated with the number of past inspections?”. The outcome of interest in our study was the reports of entomologically identified mosquito larval habitats. We defined a case as a household with a positive result for mosquito larval habitats in its most recent inspection in 2017. We defined a control as a household with a negative result for mosquito larval habitats in its most recent inspection in 2017. We applied an epidemiological "case-control” study design to nationally representative inspection data.

### Statistical analysis

We obtained the records of all household inspections carried out by NEA from 2014 to 2017. The data comprised reports of entomologically identified mosquito larvae found breeding in de-identified households, inspection dates, the nature of each inspection (outbreak or non-outbreak related), housing type, floor level of household and the community district where the household was located. We examined and excluded any de-identified inspection records which contained values that were outside the range for any variable. These included impossible values for residential floor levels and inspection time intervals. We coded the dependent variable as ‘1’ if the household was reported to have a mosquito larval habitat in the latest inspection in 2017 and ‘0’ otherwise. We modelled the total number of inspections for each household in the past 36 months from the date of the final inspection as a categorical independent variable. We aggregated the individual inspection frequencies into 4 stratums (see S1 Table). The data contained records of the time interval between the most recent two successive inspections reported on a daily timescale. We represented the effect of the time interval on the outcome of interest by categorizing the data using a 6-monthly timescale. We used values representing the registered storey of each household to create a categorical variable by coding storeys from 1 to 3 as ‘1’, 4 to 6 as ‘2’, 7 to 9 as ‘3’, 10 to 12 as ‘4’ and those higher than 12 as ‘5’. We assessed several potential confounding factors, which included the community district of residence, housing type and proximity to the ground level, the nature (outbreak/non-outbreak related) and within-year calendar timing of the most recent inspection because they could influence the outcome measure.

The patterns of independent variables for case and control groups were described separately using percentages. We made comparisons between groups using the chi-square test (χ^2^). We also used multivariable logistic regression, which is appropriate for assessing the relationship between a dependent categorical variable and multiple independent categorical or continuous variables [28]. We used the Likelihood Ratio Test (LRT) to determine the associations between the independent variables and the outcome of interest. The measure of the effect for each independent variable on the outcome of interest in the multivariable model was expressed as an adjusted odds ratio. In sensitivity analysis, we compared the stratum specific effect estimates for individual inspection frequencies (10 stratums) with the 4 stratums we used, to assess if we had aggregated the individual frequencies appropriately. We evaluated the statistical significance at the 5% level and presented chi-squared test (χ^2^) and LRT p-values, stratum specific AORs and the corresponding 95% CIs for the effects of independent variables. All analyses were performed using Stata 12.1 software (StataCorp, USA).

## Results

### Characteristics of study population

We obtained the inspection records for 589,904 households and excluded 5% (n = 29,655) that had impossible values. We analysed the remaining inspection records for 560,249 (100%) households. There were 3,205 cases (0.6%) and 557,044 controls (99.4%). Among households with a higher frequency of inspection (≥5 previous in the past 3-year period), the frequency of inspection appeared to be slightly lower for the cases compared to the controls. In contrast, the frequency of inspection was slightly higher for the cases compared to the controls at the lower inspection frequency (<5 past previous in the past 3-year period). There were 101 households with repeated reports of a mosquito larval habitats and this comprised 0.02% of all households analysed. The proportion of public apartments among cases was approximately 20% lower than that of the controls while the proportion of landed homes was about 20% higher for cases than for the controls. Among cases, the proportion of households with reports of mosquito larval breeding habitats appeared to decline with an increasing vertical distance from the ground level, while this distinction among controls was less clear. There were some differences in the spatial characteristics and the calendar month of the most recent inspections carried out between both groups (p<0.001). The characteristics of each group are summarised in Table 1.

**Table 1:** Characteristics of the study population in Singapore, 2014 to 2017.

Description of Variables and Categories	Cases (n = 3,205)	Controls (n = 557,044)	χ^2^ p-value
**Number of past inspections**			
9 to 10	74 (2.3%)	20,611 (3.7%)	<0.001
5 to 8	652 (20.3%)	126,608 (22.7%)	
1 to 4	2,017 (62.9%)	335,475 (60.2%)	
0	462 (14.4%)	74,350 (13.4%)	
**Duration between the two most recent inspections**			
<6 months	1,097 (34.2%)	199,350 (35.8%)	0.007
≤6 to <12 months	739 (23.1%)	126,974 (22.8%)	
≤12 to <18 months	405 (12.6%)	71,021 (12.8%)	
≤18 to <24 months	222 (6.9%)	41,754 (7.5%)	
≤24 to <30 months	140 (4.4%)	25,259 (4.5%)	
≤30 to <36 months	140 (4.4%)	18,336 (3.3%)	
≥36 months	462 (14.4%)	74,350 (13.4%)	
**Outcome of immediate previous inspection**			
No mosquito larval habitat reported	3,104 (96.9%)	554,525 (99.6%)	<0.001
Mosquito larval habitat reported	101 (3.2%)	2,519 (0.5%)	
**Nature of most recent inspection**			
Non-outbreak related	2,964 (92.5%)	517,925 (93.0%)	0.272
Outbreak related	241 (7.5%)	39,119 (7.0%)	
**Housing type**			
Public apartment	1,947 (60.8%)	450,054 (80.8%)	<0.001
Private apartment	405 (12.6%)	72,581 (13.0%)	
Landed house	853 (26.6%)	34,409 (6.2%)	
**Housing floor level**			
Located within 1^st^ to 3^rd^ storey	2,132 (66.5%)	224,665 (40.3%)	<0.001
Located within 4^th^ to 6^th^ storey	347 (10.8%)	80,654 (14.5%)	
Located within 7^th^ to 9^th^ storey	267 (8.3%)	76,355 (13.7%)	
Located within 10^th^ to 12^th^ storey	278 (8.7%)	97,223 (17.5%)	
Located within 12^th^ storey	181 (5.7%)	78,147 (14.0%)	
**Community district**			
Central	803 (25.1%)	148,622 (26.7%)	<0.001
North East	663 (20.7%)	112,063 (20.1%)	
North West	593 (18.5%)	118,212 (21.2%)	
South East	751 (23.4%)	93,831 (16.8%)	
South West	395 (12.3%)	84,316 (15.1%)	
**Calendar month of most recent inspection**			
January	143 (4.5%)	22,471 (4.0%)	<0.001
February	122 (3.8%)	27,896 (5.0%)	
March	167 (5.2%)	37,136 (6.7%)	
April	244 (7.6%)	29,873 (5.4%)	
May	229 (7.2%)	45,987 (8.3%)	
June	211 (6.6%)	42,463 (7.6%)	
July	244 (7.6%)	52,772 (9.5%)	
August	229 (7.2%)	57,411 (10.3%)	
September	283 (8.8%)	62,312 (11.2%)	
October	396 (12.4%)	64,124 (11.5%)	
November	530 (16.5%)	65,381 (11.7%)	
December	407 (12.7%)	49,218 (8.8%)	

### Multivariable regression analysis

**Fig 2:**
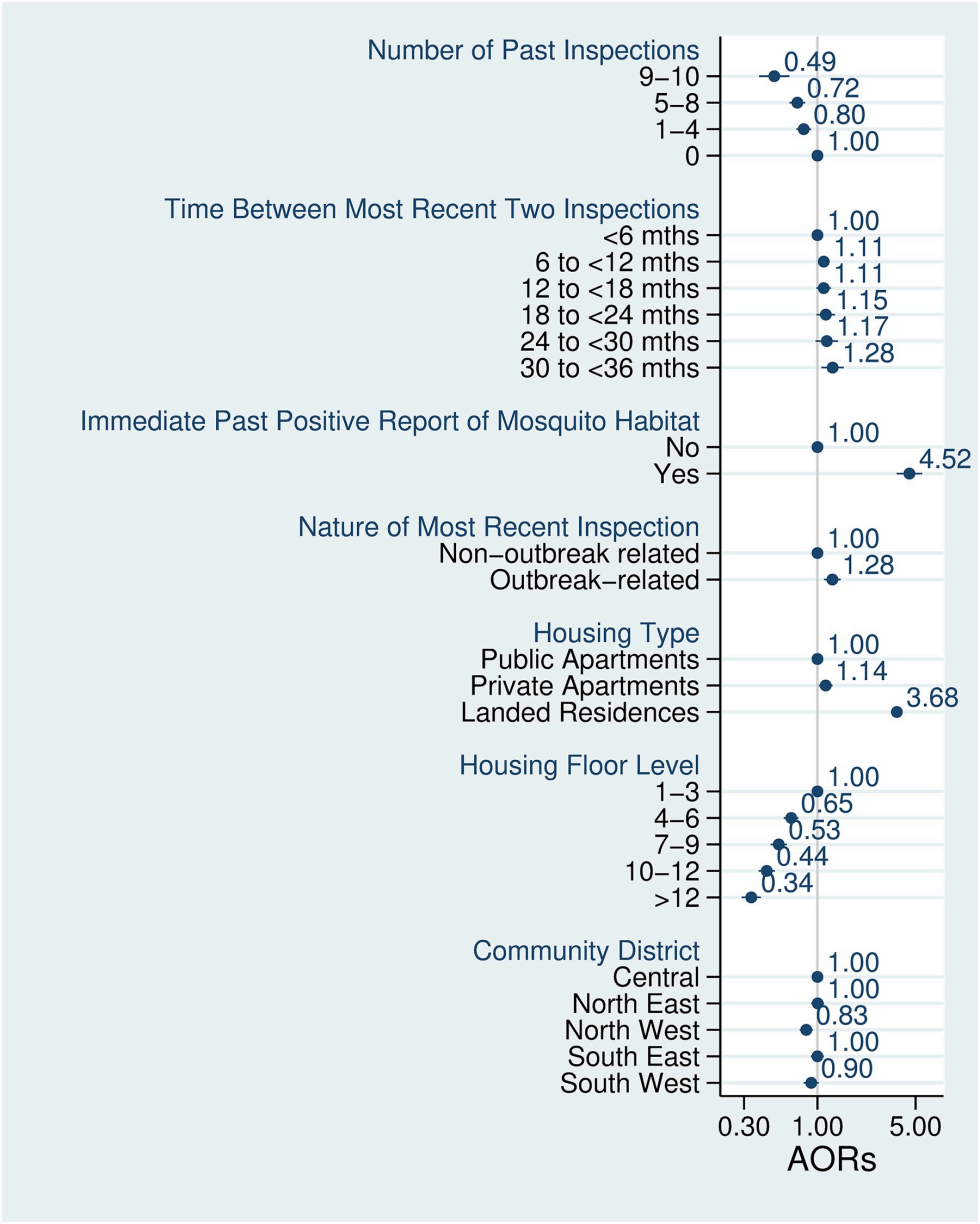
Adjusted ORs for factors associated with households reported with mosquito larval habitats in Singapore, 2017. The solid circles indicate the point estimates and the horizontal navy blue lines indicate the 95% confidence intervals for those estimates. The vertical grey line indicates the null value of 1.00. Reference categories are indicated with a value of 1.00.

For the univariate analysis, the odds of the reported habitats in households that had 9 to 10 past inspections over the 36-month period (an average of 3 inspections per annum) was 0.58 (95% CI: 0.45 to 0.74) compared to that of households that did not have any inspections (see S2 Table). After adjusting for the effects of potential confounders, the frequency of 9 to 10 previous inspections remained protective 0.49 (95% CI: 0.38 to 0.63) (Fig 2). Inspections exhibited a protective but reduced effect on the reported mosquito larval habitats at lower frequencies [AOR: 0.72 (95% CI: 0.63 to 0.82) for households with 5 to 8 previous inspections, AOR: 0.80 (95% CI: 0.71 to 0.90) for households with 1 to 4 past inspections]. These results were consistent with the individual inspection frequency-specific estimates obtained in the sensitivity analysis (see S3 Table and S1 Fig).

When the two most recent successive inspections were carried out 30 to 36 months apart, there were increased odds of mosquito larval habitat reports in households (AOR: 1.28, 95% CI: 1.06 to 1.56) compared to those carried out within 6 months. The direction and the magnitude of the effect for other time intervals on the reports of mosquito habitats were similar, though statistically significant only for the 6- to 12-month interval (AOR: 1.11, 95% CI: 1.01 to 1.22). We observed a more than four-fold increase in the odds of mosquito larval habitat reports in households that had a report in the immediate previous inspection compared to those that did not have one (AOR: 4.52, 95% CI: 3.67 to 5.56).

Outbreak related inspections were more likely to report mosquito larval habitats than routine ones (AOR: 1.28, 95% CI: 1.12 to 1.46). Among the three classes of household types, landed houses had the highest odds of mosquito larval habitat reports while private apartments had slightly higher odds compared to public apartments. We observed a clear trend with the odds of mosquito larval habitat reports decreasing in households with increasing vertical distance from the ground level. Compared to those in the Central community district, reports of mosquito larval habitats in households located in the North West district were less likely (AOR: 0.83, 95% CI: 0.75 to 0.93). The association between the calendar month of inspection and reported mosquito larval habitats was inconsistent—with higher odds in some months and lower odds in others relative to January (see S4 Table).

## Discussion

To our knowledge, this is the first large scale, nationally representative study that examined the effect of inspections on reported mosquito larval habitats in households. After adjusting for the effects of potential confounders, we found that households that were inspected more often were less likely to be associated with positive reports of mosquito larval habitats. The propensity for mosquito larval habitats in households that averaged three inspections per annum over a 3-year period was half that of those that were not inspected at all. Inspections remained protective at lower frequencies, though to a lesser degree. This study strengthens the evidence for the use of household inspections as an effective vector control intervention.

In Singapore, public health inspectors carry out inspections of accessible homes in the presence of at least one occupant. The occupant accompanies the inspector throughout the inspection process and may acquire knowledge of the specific locations where water is more likely to stagnate and become conducive to the growth of mosquito larvae. General reminders by the health inspector, along with recommendations to address specific larval habitats are common during the inspection process and reinforce occupant knowledge. Previous studies have reported an improvement in knowledge and the adoption of best practices for the reduction of *Aedes aegypti* breeding sites after education activities [29–31]. Health inspectors only apply Temephos larvicide to containers positive for mosquito larvae and occupants are taught to regular remove any standing water thereafter. Therefore, reductions in the mosquito population are more likely to be attributable to the frequency of standing water removal rather than the one-time application of larvicides. We suggest that inspections may increase occupant knowledge and awareness and thus contribute in part to improved household practices that reduce entomological activity. The detection of larval habitats during an inspection results in the issuance of a punitive fine to the household occupant [32]. A study conducted in Brazil reported that government pressure coupled with warnings and fines were the most effective measure in reducing entomological breeding sites in commercial establishments at high risk for *Aedes aegypti* propagation [33]. We also suggest that the perceived risk of punitive fines may also influence household practices in part, though additional research is required to determine the independent effects of deterrence and knowledge.

We found clear evidence that longer durations between successive inspections increased the risk of positive mosquito larval habitat reports in households. This finding suggests that delaying subsequent inspections may erode the protective effects from previous inspections. Health authorities should also consider higher and regularly spaced inspection frequencies as a strategy for reducing entomological activity in households. Desired inspection frequencies must however reflect the balance between the economic costs and anticipated public health benefits.

Not surprisingly, we found that outbreak-related household inspections were positively associated with reports of mosquito larval habitats. Increased reports may be due to elevated vector activity within disease outbreak areas. The NEA intensifies its household inspections in dengue outbreak areas to quickly seek out and destroy sources of vector activity [34, 35]. The observed association may be in part due to the increased motivation of health inspectors to uncover and eliminate sources of mosquito larval habitats in disease outbreak areas. Since the intention of inspections during an outbreak is to identify and eliminate as many sources of mosquito activity as possible, this study finding is reassuring and reflects the ability of inspections to identify more household sources of mosquito activity in residential areas with elevated dengue transmission.

We also observed the influence of spatial and household-level characteristics on the reporting of mosquito larval habitats in households in Singapore. There were spatial differences in the reports of mosquito larval habitats among the community districts of residence, with lower levels observed in the western districts compared to the others, though this was only significant for the North-western district. This appears to correspond with historical spatial trends for dengue reports in Singapore that indicate that the western community districts had comparatively lower levels of reported dengue infections [36].

Previous studies have reported on the influence of household-level and environmental characteristics on the entomological activity in homes. A study carried out in Sant Cugat (Spain) reported that environmental characteristics such as the presence of solid waste, scuppers, construction sites, and stacked gardening or building materials were positively associated with the presence of *Aedes albopictus* mosquito larval habitats in households [37]. Another study carried out in Machala (Ecuador) reported that factors such as the condition of the house and patio, water storage practices and lack of access to piped water were positively associated with the presence of *Aedes aegypti* pupal habitats in households [38]. In our study, we found that reports of mosquito larval habitats were more likely in landed households compared to apartments. Our observations were in agreement with the findings from an earlier study conducted in Singapore [39]. Landed households generally have outdoor paved and turf areas that are directly exposed to the weather elements. Vegetation in these areas may provide refuge for adult mosquitoes during warmer weather. Plants and containers placed in these areas may accumulate stagnant water and become sources of mosquito activity. Relative to apartments, landed households have larger living spaces and the propensity for a higher number of mosquito larval habitat reports may be as a result the larger number of sites for stagnant water to develop into larval breeding habitats.

In our study, the proclivity for reports of mosquito larval habitats in households declined with increasing vertical distance from the ground level. Households located on lower floors are proximate to natural vegetation, discarded receptacles and street level storm water drains that can accumulate stagnant water due to rainfall. These natural and artificial containers may become conducive mosquito breeding sites if the stagnant water within is not removed. Our study findings are consistent with that from another study on the use of gravitraps, which reported lower *Aedes aegypti* activity on higher floors of high-rise buildings [40]. A previous study analysing the data collected from ovitraps deployed in high-rise apartment blocks in Malaysia reported the presence *Aedes aegypti* mosquito larvae on most floors though the negative relationship between larvae presence and floor level was not statistically significant [41]. The difference in the size of the studies may have contributed to the difference in study findings.

A previous study advocated the merits of spatial-temporal dengue forecasts in targeting vector control activities at the neighbourhood level [42]. While our main study findings point to the benefits of increasing inspection frequencies for reducing entomological activity in households, practical resource limitations restrict its adoption across all households in any identified neighbourhood. The observed dependence of mosquito larval habitat reports on the spatial, temporal and household-level characteristics from our study reinforce the need to adopt a risk-based approach in executing vector control at the household level. We recommend accounting for such factors when prioritizing the allocation of limited vector control resources.

In our study, we found that immediate previous reports of mosquito larval habitats were positively associated with subsequent reports of habitats in the most recent inspections. Though the estimated strength of this association was high, the proportion of households with repeated reports was extremely low. This may be due to success of the NEA’s household inspection programme and public education efforts. Nevertheless, public health inspectors from the NEA seeking to eliminate household sources of entomological activity could expeditiously prioritize their vector control efforts in households with past reports. Given that the report of a mosquito larval habitat is a proxy for a punitive fine received by the household, this finding may suggest that the present penalty regime alone is insufficient in incentivizing positive behaviour in a small number of households. Additional research to determine the association between the characteristics of such households and their occupants with sustained entomological activity is required. Discovering the factors associated with a propensity for entomological activity in households may inform the design and choice of strategies aimed at reducing repeated reports of mosquito larval habitats.

### Study strengths and limitations

We analysed a large and nationally representative (n = 560,249) dataset. We included 95% of all household inspection records in our data analysis, thus greatly minimizing selection bias. We used a well-defined outcome measure–entomologically confirmed mosquito larval habitats, and thus the potential for case misclassification was low. In the absence of data on actual fines meted out to each household, we used outcomes of past inspections as a proxy in order to examine their subsequent effect on reported mosquito larval habitats. To the best of the authors’ knowledge, the NEA infrequently accedes to appeals for fine waivers related to such reports. Therefore, the effect of fines is likely to be similar to our estimate for the effect of past reports of mosquito habitats on subsequent inspection outcomes. The likelihood of detecting statistically significant findings in this highly powered study was high. However, given the large number of households inspected annually (>550,000), even a 10% change in risk may translate into a substantial change in the absolute number of positive mosquito larval habitats reported. The NEA did not collect household-level information to assess the impact of localized community initiatives on entomological activity in households. We were thus unable to account for their independent effects on reports of mosquito larval habitats in households. Additional studies examining the effectiveness of such community initiatives are recommended.

### Conclusions

Vector control remains an important strategy in arboviral disease control. While inspections seek to reduce sources of arboviral vectors, evidence of their effectiveness is limited. Ours is the first nationally representative study to demonstrate the protective effect of inspections on entomological activity in households, thus providing evidence of its effectiveness as a vector control intervention. Our main study finding is reassuring to health authorities that intend to use or continue with inspections to reduce entomological activity in households. We recommend that arboviral disease control programmes account for spatial, temporal and household-level characteristics in prioritizing vector control efforts. Research on alternative strategies may help address recurrent reports of mosquito larval habitats in households, though periodic assessments to ensure their effectiveness may be necessary.

## Supporting information

S1 Checklist STROBE checklist. (DOC)

S1 Table Descriptive analysis of inspection frequencies according to cases and controls. (DOCX)

S2 Table Results of univariate analysis. (DOCX)

S3 Table Results of multivariable regression using stratum specific estimates corresponding to each of the 10 inspection frequencies. (DOCX)

S4 Table Results of final multivariable regression analysis. (DOCX)

S1 Fig Adjusted ORs for factors associated with households reported with mosquito larval habitats in Singapore, 2017. Stratum specific effect estimates corresponding to each inspection frequency (i.e. 1 to 10) were obtained. The solid circles indicate the point estimates and the horizontal navy blue lines indicate the 95% confidence intervals for those estimates. The vertical grey line indicates the null value of 1.00. Reference categories are indicated with a value of 1.00.
(DOCX)

S2 Fig Regression output indicating collinearity for inspection intervals exceeding 36 months. (DOCX)
