# Supplementary material for: The effectiveness of inspections on reported mosquito larval habitats in households: A case-control study
Source: PLoS Negl Trop Dis. 2019 Jun 26;13(6):e0007492. doi: 10.1371/journal.pntd.0007492 (PMC6615626; doi:10.1371/journal.pntd.0007492)
Supplement: S1 Table — (DOCX) [file pntd.0007492.s002.docx]

**S1 Table. Descriptive analysis of inspection frequencies according to cases and controls.**

| **Description of Variables and Categories** | **Cases (n=3,205)** | **Controls (n=557,044)** | χ^2^ **p-value** |
| --- | --- | --- | --- |
| **Number of past inspections** |  |  | <0.001 |
| 0 | 462 (14.4%) | 74,350 (13.4%) |  |
| 1 | 561 (17.5%) | 92,167 (16.6%) |  |
| 2 | 567 (17.7%) | 92,808 (16.7%) |  |
| 3 | 488 (15.2%) | 82,626 (14.8%) |  |
| 4 | 401 (12.5%) | 67,874 (12.2%) |  |
| 5 | 262 (8.2%) | 51,071 (9.2%) |  |
| 6 | 191 (6.0%) | 36,097 (6.5%) |  |
| 7 | 122 (3.8%) | 24,073 (4.3%) |  |
| 8 | 77 (2.4%) | 15,367 (2.8%) |  |
| 9 | 28 (0.9%) | 9,117 (1.6%) |  |
| 10 | 46 (1.4%) | 11,494 (2.1%) |  |
