# Supplementary material for: The effectiveness of inspections on reported mosquito larval habitats in households: A case-control study
Source: PLoS Negl Trop Dis. 2019 Jun 26;13(6):e0007492. doi: 10.1371/journal.pntd.0007492 (PMC6615626; doi:10.1371/journal.pntd.0007492)
Supplement: S2 Table — (DOCX) [file pntd.0007492.s003.docx]

**S2 Table. Results of univariate analysis.**

| **Characteristics** | **Odds Ratio** | **95% CI** | **Wald test  p-value** |
| --- | --- | --- | --- |
| **Number of past inspections** |  |  |  |
| 9 to 10 | 0.58 | 0.45 - 0.74 | <0.001 |
| 5 to 8 | 0.83 | 0.74 - 0.93 | 0.002 |
| 1 to 4 | 0.97 | 0.87 - 1.07 | 0.524 |
| 0 | Referent |  |  |
| **Duration between the two most recent inspections** |  |  |  |
| <6 months | Referent |  |  |
| ≤6 to <12 months | 1.06 | 0.96 - 1.16 | 0.240 |
| ≤12 to <18 months | 1.04 | 0.92 - 1.16 | 0.541 |
| ≤18 to <24 months | 0.97 | 0.84 - 1.12 | 0.641 |
| ≤24 to <30 months | 1.01 | 0.84 - 1.20 | 0.936 |
| ≤30 to <36 months | 1.39 | 1.16 - 1.66 | <0.001 |
| ≥36 months | 1.13 | 1.01 - 1.26 | 0.029 |
| **Outcome of immediate previous inspection** |  |  |  |
| No mosquito larval habitat reported | Referent |  |  |
| Mosquito larval habitat reported | 7.16 | 5.85 - 8.77 | <0.001 |
| **Nature of most recent inspection** |  |  |  |
| Non-outbreak related | Referent |  |  |
| Outbreak related | 1.08 | 0.94 - 1.23 | 0.273 |
| **Household type** |  |  |  |
| Public apartment | Referent |  |  |
| Private apartment | 1.29 | 1.16 - 1.44 | <0.001 |
| Landed house | 5.73 | 5.28 - 6.22 | <0.001 |
| **Housing floor level** |  |  |  |
| Located within 1^st^ to 3^rd^ storey | Referent |  |  |
| Located within 4^th^ to 6^th^ storey | 0.45 | 0.40 - 0.51 | <0.001 |
| Located within 7^th^ to 9^th^ storey | 0.37 | 0.32 - 0.42 | <0.001 |
| Located within 10^th^ to 12^th^ storey | 0.30 | 0.27 - 0.34 | <0.001 |
| Located above 12^th^ storey | 0.24 | 0.21 - 0.28 | <0.001 |
| **Community district** |  |  |  |
| Central | Referent |  |  |
| North East | 1.10 | 0.99 - 1.21 | 0.085 |
| North West | 0.93 | 0.83 - 1.03 | 0.171 |
| South East | 1.48 | 1.34 - 1.64 | <0.001 |
| South West | 0.87 | 0.77 - 0.98 | 0.021 |
| **Calendar month of most recent inspection** |  |  |  |
| January | Referent |  |  |
| February | 0.69 | 0.54 - 0.88 | 0.002 |
| March | 0.71 | 0.56 - 0.88 | 0.002 |
| April | 1.28 | 1.04 - 1.58 | 0.018 |
| May | 0.78 | 0.63 - 0.96 | 0.022 |
| June | 0.78 | 0.63 - 0.97 | 0.023 |
| July | 0.73 | 0.59 - 0.89 | 0.002 |
| August | 0.63 | 0.51 - 0.77 | 0.000 |
| September | 0.71 | 0.58 - 0.87 | 0.001 |
| October | 0.97 | 0.80 - 1.18 | 0.759 |
| November | 1.27 | 1.06 - 1.53 | 0.010 |
| December | 1.30 | 1.07 - 1.57 | 0.007 |
