# Supplementary material for: The effectiveness of inspections on reported mosquito larval habitats in households: A case-control study
Source: PLoS Negl Trop Dis. 2019 Jun 26;13(6):e0007492. doi: 10.1371/journal.pntd.0007492 (PMC6615626; doi:10.1371/journal.pntd.0007492)
Supplement: S4 Table — (DOCX) [file pntd.0007492.s005.docx]

**S4 Table. Results of final multivariable regression analysis.**

| **Characteristics** | **AOR** | **95% CI** | **LRT p-value** |
| --- | --- | --- | --- |
| **Number of past inspections** |  |  | <0.001 |
| 9 to 10 | 0.49 | 0.38 - 0.63 |  |
| 5 to 8 | 0.72 | 0.63 - 0.82 |  |
| 1 to 4 | 0.80 | 0.71 - 0.90 |  |
| 0 | Referent |  |  |
| **Duration between the two most recent inspections** |  |  | 0.047 |
| <6 months | Referent |  |  |
| ≤6 to <12 months | 1.11 | 1.01 - 1.22 |  |
| ≤12 to <18 months | 1.11 | 0.98 - 1.25 |  |
| ≤18 to <24 months | 1.15 | 0.98 - 1.34 |  |
| ≤24 to <30 months | 1.17 | 0.97 - 1.40 |  |
| ≤30 to <36 months | 1.28 | 1.06 - 1.56 |  |
| **Outcome of immediate previous inspection** |  |  | <0.001 |
| No mosquito larval habitat reported | Referent |  |  |
| Mosquito larval habitat reported | 4.52 | 3.67 - 5.56 |  |
| **Nature of most recent inspection** |  |  | <0.001 |
| Non-outbreak related | Referent |  |  |
| Outbreak related | 1.28 | 1.12 -1.46 |  |
| **Household type** |  |  | <0.001 |
| Public apartment | Referent |  |  |
| Private apartment | 1.14 | 1.02 - 1.08 |  |
| Landed house | 3.68 | 3.34 - 4.04 |  |
| **Housing floor level** |  |  | <0.001 |
| Located within 1^st^ to 3^rd^ storey | Referent |  |  |
| Located within 4^th^ to 6^th^ storey | 0.65 | 0.58 - 0.73 |  |
| Located within 7^th^ to 9^th^ storey | 0.53 | 0.46 - 0.61 |  |
| Located within 10^th^ to 12^th^ storey | 0.44 | 0.38 - 0.50 |  |
| Located above 12^th^ storey | 0.34 | 0.29 - 0.40 |  |
| **Community district** |  |  | 0.002 |
| Central | Referent |  |  |
| North East | 1.00 | 0.91 - 1.11 |  |
| North West | 0.83 | 0.75 - 0.93 |  |
| South East | 1.00 | 0.90 - 1.11 |  |
| South West | 0.90 | 0.80 - 1.02 |  |
| **Calendar month of most recent inspection** |  |  | <0.001 |
| January | Referent |  |  |
| February | 0.68 | 0.53 - 0.86 |  |
| March | 0.73 | 0.59 - 0.92 |  |
| April | 1.27 | 1.03 - 1.57 |  |
| May | 0.84 | 0.68 - 1.04 |  |
| June | 0.79 | 0.64 - 0.98 |  |
| July | 0.79 | 0.64 - 0.98 |  |
| August | 0.64 | 0.52 - 0.79 |  |
| September | 0.76 | 0.62 - 0.93 |  |
| October | 1.01 | 0.83 - 1.23 |  |
| November | 1.41 | 1.17 - 1.71 |  |
| December | 1.32 | 1.09 - 1.60 |  |
| Note: The results for the effect of the duration between the two most recent inspections exceeding 36 months were omitted due to collinearity (see S2 Fig). | | | |
