# Supplementary material for: The effectiveness of inspections on reported mosquito larval habitats in households: A case-control study
Source: PLoS Negl Trop Dis. 2019 Jun 26;13(6):e0007492. doi: 10.1371/journal.pntd.0007492 (PMC6615626; doi:10.1371/journal.pntd.0007492)
Supplement: S1 Fig — Stratum specific effect estimates corresponding to each inspection frequency (i.e. 1 to 10) were obtained. The solid circles indicate the point estimates and the horizontal navy blue lines indicate the 95% confidence intervals for those estimates. The vertical grey line indicates the null value of 1.00. Reference categories are indicated with a value of 1.00. (DOCX) [file pntd.0007492.s006.docx]

**S1 Fig. Adjusted ORs for factors associated with households reported with mosquito larval habitats in Singapore, 2017.**

Stratum specific effect estimates corresponding to each inspection frequency (i.e. 1 to 10) were obtained. The solid circles indicate the point estimates and the horizontal navy blue lines indicate the 95% confidence intervals for those estimates. The vertical grey line indicates the null value of 1.00. Reference categories are indicated with a value of 1.00.
