# Supplementary figures and images for: The effectiveness of inspections on reported mosquito larval habitats in households: A case-control study
Source: PLoS Negl Trop Dis. 2019 Jun 26;13(6):e0007492. doi: 10.1371/journal.pntd.0007492 (PMC6615626; doi:10.1371/journal.pntd.0007492)

**Fig. S2 Regression output indicating collinearity for inspection intervals exceeding 36 months**


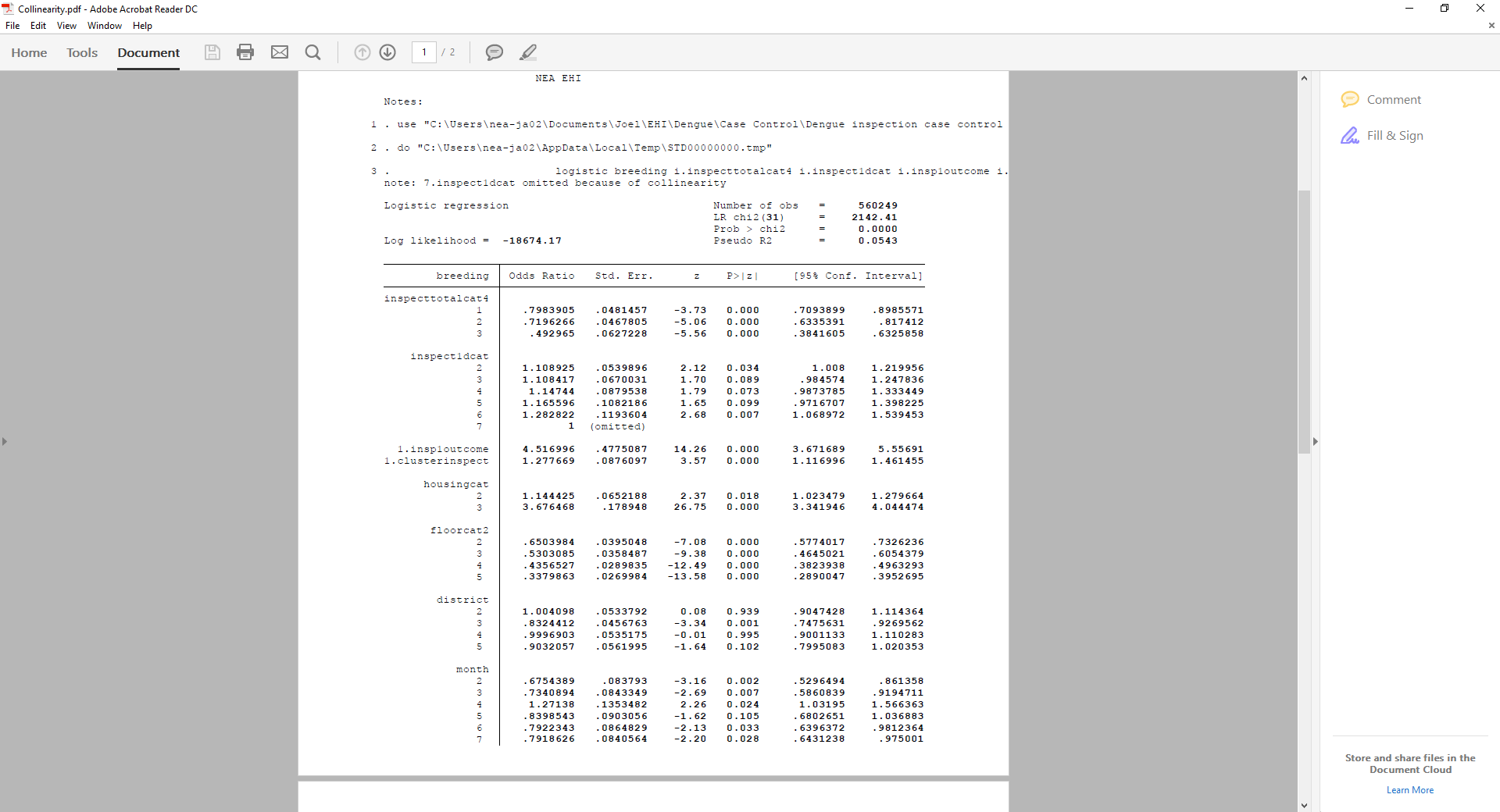

Supplement: S2 Fig — (DOCX) [file pntd.0007492.s007.docx]
